# Determination and improvement of the quality of separately collected bio-waste from households

**DOI:** 10.1177/0734242X241259895

**Published:** 2024-08-20

**Authors:** Josef Adam, Martin Wellacher, Ferozan Azizi, Alexandra Loidl, Andreas Zöscher, Franz Poschacher, Roland Pomberger, Alexia Tischberger-Aldrian

**Affiliations:** 1 Chair of Waste Processing Technology and Waste Management, Montanuniversitaet Leoben, Leoben, Austria; 2 Ingenieurbüro Wellacher e.U., Graz, Austria; 3 Holding Graz – Kommunale Dienstleistungen GmbH, Sparte Abfallwirtschaft, Graz, Austria; 4 Abfallwirtschaftsverband Mürzverband, Kapfenberg, Austria; 5 Poschacher Kompost, Kraubath, Austria

**Keywords:** Bio-waste, collection, compost, quality, impurities, pre-collection bag

## Abstract

The recycling of bio-waste from households is an essential factor in achieving the recycling quotas for municipal waste laid down by the EU. A major problem is posed by impurities in the bio-waste collected, such as plastics, metals and glass. It is virtually impossible for compost producers to produce quality-assured compost from bio-waste with an impurity content of more than 3 wt%_OS_. The draft of the new Austrian Compost Ordinance stipulates a limit of 2 wt%_OS_ of interfering substances in accepted bio-waste. A rapid measurement method has been developed and comprehensively validated for the immediate on-site checking of contaminant content at the bio-waste bin or in a vehicles. Data on the type and amount of impurities collected in the course of sorting analyses carried out over several years in 10 selected areas in Styria, Austria showed an average impurity content of 2.1 wt%_OS_. This impurity content can be considered representative for rural and urban communities in Austria. Among the interfering substances, plastics predominate, at 53%, of which pre-collection bags made of plastics form the highest proportion. A more detailed examination of pre-collection bags shows a higher proportion of use of biodegradable plastic bags, which have become more numerous in recent years in the more rural communities. In order to reduce mis-sorting, the effect of a wide variety of measures on citizens was tested in selected areas. Here, the distribution of paper bags as well as the threat of a cost increase due to special collections in combination with distribution of these bags were the methods with the greatest effect. Motivational letters and the threat of special collections, however, showed no significant result.

## Introduction

Bio-waste is the basis for quality compost, and it is crucial for a modern, functioning circular economy. This important resource is becoming increasingly important in terms of quantity and quality internationally and for the member countries of the European Union (EU). In European municipal waste generation, bio-waste from households constitutes a significant waste stream. Of the 249 million tonnes of solid municipal waste generated by the 28 EU Member States in 2017, 86 million tonnes were accounted for by separately collected bio-waste from households and waste still mixed with residual waste, that is, around 34% (Reichel, 2020). According to the European Compost Network data report, less than 40 million tonnes of municipal bio-waste are collected separately in Europe and subsequently recycled organically aerobically (composting) or anaerobically (digestion), which corresponds to a share of only 16% (European Compost Network ECN e.V., 2022).

Bio-waste is the most essential factor in achieving the recycling targets for municipal waste. In future, municipalities will have to collect more bio-waste from households in order to achieve the EU-mandated recycling rates for municipal waste of 55% (2025), 60% (2030) and 65% (2035). Member States are obliged to collect bio-waste separately for recycling or to compost it on site since 31 Dec 2023 (European Parlament, 2018).

In Austria, bio-waste has been collected separately for decades on the basis of the Ordinance on the Separate Collection of Bio-waste (Bundesgesetz, 1992). In this way, Austria has played a pioneering role within the European Union. A distinction is essentially made between a collection system and a delivery system in terms of the separate collection of municipal waste in Austria, such as mixed municipal waste, bulky waste, bio-waste, waste paper, waste glass, lightweight packaging and metal waste. As a rule, mixed municipal waste, lightweight packaging and bio-waste are subject to a collection system, that is, the respective bins are collected directly from the households. A total of 4,630,711 tonnes of municipal waste was generated by households in 2020. The volume of bio-waste amounted to 1,137,410 tonnes or 24.6%. On average, 128 kg per inhabitant per year is collected, ranging from 50 kg/(cap.*a) in Vienna to 224 kg/(cap.*a) in Burgenland, depending on the connection rate or the share of self-composting. The federal state of Styria has a rate of 110 kg/(cap.*a) (Federal Ministry for Climate Action, Environment, Energy, Mobility, Innovation and Technology, 2023). In 2019, 10,207,873 tonnes of bio-waste were collected separately in the 16 federal states in Germany. The average was 109 kg per inhabitant per year, with a range from 32 kg/(cap.*a) in Berlin to 172 kg/(cap.*a) in Rhineland-Palatinate (Bund/Länder-Arbeitsgemeinschaft Abfall (LAGA) 2022).

A waste management issue that has become more important in recent years is the problem of contaminants in compost. The accumulation of unwanted materials such as plastics, metals and glass in bio-waste collected from households is increasingly jeopardising its recycling this into compost. Complaints from customers about the visible plastic content are increasing. As a result, the marketing revenues for compost are decreasing, the transfer costs to the composting plant operators are increasing for the municipalities and more batches are rejected because they have too many impurities. In Styria, for example, individual composting plant operators have terminated their takeover contracts with municipalities due to excessive levels of contaminants. In some cases, the municipalities have to at least undertake to take back contaminants separated by the composting plant operator, which leads to additional costs.

Against this background, the topic of contaminant management and improving the quality of separately collected bio-waste has been becoming more important for years. In Germany, batch analyses carried out by the Bundesgüte Gemeinschaft Kompost show an impurity content of between less than 1 wt%_OS_ and around 5 wt%_OS_ by weight in bio-waste delivered (Bundesgütegemeinschaft Kompost e.V., 2018/2019).

Other analyses of municipal deliveries revealed impurity contents of between 1.4 and 9.1 wt%_OS_ (Jörg, 2019). On average, impurity contents of 2–3 wt%_OS_ by weight are assumed (Kehres, 2021). Analyses by the Italian Compost and Biogas Association (CIC) show that kitchen waste in particular contains 4.9 wt%_OS_ by weight of contaminants, of which plastic bags and packaging account for the largest share, at 54.9% (Ricci and Centemero, 2018).

In Germany in particular, numerous projects and initiatives to improve the quality of bio-waste in household collections have been successfully implemented in district organisations and local authorities. In one district, for example, a consumer information programme with a paper bag and a voucher for the free purchase of five additional bags has led to the halving of the impurity content from 3.5 by weight to 1.75 wt%_OS_ by weight. By monitoring the amounts using a geodata-based mobile phone app, the impurity content could be significantly reduced again, from 1.75 to 0.4 wt%_Os_ (Idelmann et al., 2022).

Other initiatives range from classic public relations work, such as information in local newspapers, radio adverts and guided tours of the composting plant, to individual environmental education in schools and household inspections before the organic waste bin is emptied, combined with possible penalties (Habermann, 2022).

A cycle-oriented approach and an improvement in individual processes are essential to produce quality-assured compost from bio-waste. This article deals with the first process in the chain: the collection and the improvement of the quality of bio-waste in household collection in selected municipalities or areas in the Austrian province of Styria.

From a quality point of view, it is hardly possible for compost producers to produce quality-assured compost from bio-waste with an impurity content of more than 3 wt%_OS_ that complies with the applicable limits and is visually free of impurities. In addition, during the processing of raw compost, increasingly fine screening to remove impurities leads to a discrepancy between the impurity content in the compost and the loss of good material in the screen overflow (Kehres, 2017).

The draft of a new Austrian Compost Ordinance aims to professionalise composting and stipulates a maximum limit of 2 wt%_OS_ of unwanted substances in bio-waste from household collection accepted by composting plants (Federal Ministry for Climate Action, Environment, Energy, Mobility, Innovation and Technology, 2021).

There is therefore a need for a simple and rapid determination of the contaminant content when accepting and handing over bio-waste, both from the citizen to the municipality and from the municipality to the compost producer. In addition, individual municipalities plan separate routes or special collections for dirtier than normal bio-waste bins, and this requires proof to be provided to the customer concerned.

Currently, the determination of impurities by manually sorting a 25 kg organic waste bin contents takes about 25 minutes, and about 4 hours are needed for one person to sort the contents of a 500 kg collection vehicle (Bundesgütegemeinschaft Kompost e.V., 2021). This is too much effort in order to determine whether the content is too high so that specific measures can be taken, although isolated approaches to speeding the determination of interfering substances have been described (Bauer, 2017; Giani and Pretz, 2015; Kern et al., 2017).

The collection of bio-waste directly at the point of origin is the first step in the process towards influencing quality. Time and again, municipalities have tested various measures to change citizens’ behaviour (Arbeck et al., 2022; Idelmann et al., 2022).

It is known that contamination with impurities is higher in settlement structures with a greater density of inhabitants, that is, in blocks of flats rather than in single-family houses (Wellacher and Krenn, 2017).

Due to different settlement structures in urban and rural areas, various pre-collection bags are used for the collection of bio-waste. The use of pre-collection bags is particularly helpful in the case of blocks of flats, where the route to the bio-waste bins is longer and the possibility of self-composting is not available. One topic that has been widely and intensively discussed in German-speaking countries is the type of pre-collection aid, in particular whether biodegradable plastic bags are permitted for this purpose or are also considered to be interfering materials in bio-waste. An expert paper from the Austrian Water and Waste Management Association (ÖWAV) shows that pre-collection bags made of plastics, irrespective of the raw material basis, are to be regarded as interfering materials, as they have to be sorted out in the further composting process with a high technical, financial and/or time cost before they can be fed into an energy recycling process (ÖWAV, 2021).

In terms of application, the range of bags on offer commercially is also relevant from the consumer’s point of view, together with what range and strategy the individual municipality is pursuing. There are also expectations regarding handling, odour nuisance, tear resistance and costs.

Considering this background, the following objectives were set for this study: (1) to obtain reliable data material on the type and quantity of interfering substances in selected areas; (2) to develop a rapid measurement method to make it easier to measure the impurity content; (3) to investigate the effect of individual measures for changing citizens’ behaviour or reducing misdirected waste in the disposal of bio-waste, focusing on blocks of flats, including the long-term effects and (4) to describe usage behaviour for the most common types of pre-collection bags.

## Materials and methods

The methods used serve to determine the impurity content in unprocessed solid bio-waste delivered to composting plant operators. The aim of the methods described in this chapter is to determine the impurity content of bio-waste at bio-bin and vehicle level and to measure the effectiveness of individual measures to improve the quality of the contents of individual bio-waste bins. These are mainly qualitative determination methods.

### Characterising the material

The waste stream considered was bio-waste from private households in the province of Styria, Austria, from 10 different municipalities or areas, which can be regarded as representative for rural and urban areas in Austria. Bio-waste is collected in containers (bio-waste bin) with a capacity of 120 and 240 l. The volume of the collection containers is adapted to the use of the respective property and depends on the number of residential units or residents, the volume of waste and the disposal interval. The size of the containers has no influence on the contents and the quality is to be regarded as equivalent.

The contaminant load of bio-waste collected is very uneven and subject to strong annual fluctuations. Taking the example of a compost producer in Styria (Kompostproduzent Steiermark, 2020) from the year 2020, the impurity content varies between 2.2 wt%_OS_ in May and 7.5 wt%_OS_ in January with an annual average of 3.7 wt%_OS_ and an average content of 3.0 wt%_OS_ in the warmer months versus 5.9 wt%_OS_ in the colder months. Such seasonal fluctuations of the impurity content have also been described for German municipalities (Jansen et al., 2020).

The figures refer to weight percent and are abbreviated to wt%. The abbreviation _OS_ stands for original substance. This refers to the sample in its original state at the time of collection, including the water content.

Bio-waste essentially consists of organic kitchen waste and garden waste. According to Article 3 No. 4 of the EU Waste Framework Directive, “bio-waste” means biodegradable garden and park waste, food and kitchen waste from households, offices, restaurants, wholesalers, canteens, caterers and from the retail trade, as well as comparable waste from food processing plants (European Parlament, 2018).

### Determining the impurity content in bio-waste

The method specifications of the German Federal Compost Quality Association (Bundesgütegemeinschaft Kompost) are used as the basis for analysis for manual sorting and the rapid measurement method. These are methods for determining the quality of solid bio-waste, whose usefulness has been proven in practice in districts and municipalities. Specific sampling concepts for different areas were created based on this. The manual sorting includes categories that are customary in the industry.

#### Reference methods: Manual sorting

In order to obtain reliable data on the type and quantity of interfering substances in bio-waste, samples were taken and 21 manual sorting analyses were subsequently carried out, divided into 10 different areas in Styria over a period of about 3 years.

The investigations were carried out following the area analysis of the Bundesgütegemeinschaft Kompost (Kehres, 2018) or the batch analysis for determining the impurity content (Kehres and Thelen-Jüngling, 2021).

For each area analysis, two sampling units of at least 1 m^3^ and at least 250 kg each were defined in advance as the collection and sorting quantity. Based on a bulk density of 0.25 kg/l, 20 properties were set as the minimum quantity for collection per area (cf. Table 1). The collection was carried out mainly by collecting bio-waste bins with 120 or 240 l volume at the selected properties, distributed over the respective area. In the case of manual sorting Nos. 13–17, the analysis of whole vehicle contents from entire routes was carried out. In areas where the total amount of batches collected was less than 2 × 250 kg or fewer sites were analysed, access to individual bio-waste bins was sometimes not possible, the fill level was unpredictably low or the bin was empty (see sorting numbers 2, 7, 11, 12 and 19).The sorting depth (i.e. the number of sorted fractions) for the individual analyses comprised 5−16 fractions, depending on the objective, which were then combined into five to six groups for a clearer comparison and presentation of results (cf Table 1).

**Table 1.** Sorted impurity groups and impurity fractions.

Impurity groups		Impurity fractions	
Designation	Group	No.	Fraction
A	Plastic bags	1	Plastic bags packaging (not biodegradable)
B	Plastic bags degradable	2	Plastic bags biodegradable
C	Plastic, general excl. plastic bags	3	Plastics packaging
		4	Plastics non-packaging/other
D	Glass, ceramics, metal	5	Glass packaging
		6	Glass non-packaging
		7	Metal packaging
		8	Metal non-packaging
E	Compounds/other	9	Composites packaging
		10	Composites non-packaging
		11	Other foreign substances/textiles/minerals
		12	Pollutants (hazardous waste)
Non-interfering group		Not to be evaluated as an interfering substance	
F	Non-interfering materials	13	Paper bags
		14	Paper other
		15	Garden waste/stones/bricks
		16	Biogoods

The following sorting rules were observed:

1. Bags were to be emptied completely and the contents sorted.
2. Stickers and adhesives on plastic bags were also included in the weighing.
3. Filled bottles were to be emptied, and their contents were counted as organic waste.
4. Composites, such as plastic paper films from the sausage counter, are not to be separated (and sorted as composite packaging).
5. Green waste, paper, natural stones or bricks are bio-waste.
6. Soil is a biogoods.

In order to gain knowledge about the pre-collection bags used, a piece count of plastic, biodegradable plastic and paper bags used for the pre-collection of bio-waste was carried out during the manual sorting analyses^13–21^.

Although manual sorting analysis is among the most accurate, it is the most time-consuming and labour-intensive method to determine the impurity content in bio-waste. This method is also impractical in the field for direct on-site assessment of a specific household’s associated bio-waste bins or of a collected tour at the vehicle level.

For this reason, a faster alternative method for determining the impurity content (rapid measurement method) was developed and extensively validated (cf. sections ‘Rapid measurement method for bio-waste bins (bin level)’ and ‘Rapid measurement method for routes (vehicle level)’). This is a visual method in which the number of superficially visible impurities in a defined quantity of bio-waste is determined by simple counting. This can be done at both bin and vehicle level.

#### Rapid measurement method for bio-waste bins (bin level)

This method was investigated in more detail for quick determination of the impurity content in the collection of individual organic bio-waste tonnes, in order to assign a property to a dirty collection route (dirty route) if necessary once a certain limit value has been exceeded. In the case of a dirty route, bio-waste is disposed of via the residual waste route at the cost of residual waste.

A uniform system and traceability are necessary to confirm to the property owner why a collection has been excluded. The values measured in the visual counting and sorting were related and the correlation is determined by means of a regression line. In the visual counting method, the number of visible interfering materials was visually recorded by an employee after the collected organic waste material had been poured onto the sorting table. The value in pieces measured was related to the filling volume determined and calculated as pieces per 12 l bulk volume.

The correlation between actual impurity content in %_OS_ on the *y*-axis and volume based on pieces/12 l (*x*-axis) of individual bin contents was compared for the studies in 2020. In 2021, a comparison was made between the amount of impurities in relation to the surface area in pieces/m^2^. The individual bio-waste bins were emptied, the material was distributed by area, and the impurities were again counted visually.

**Figure 1.**
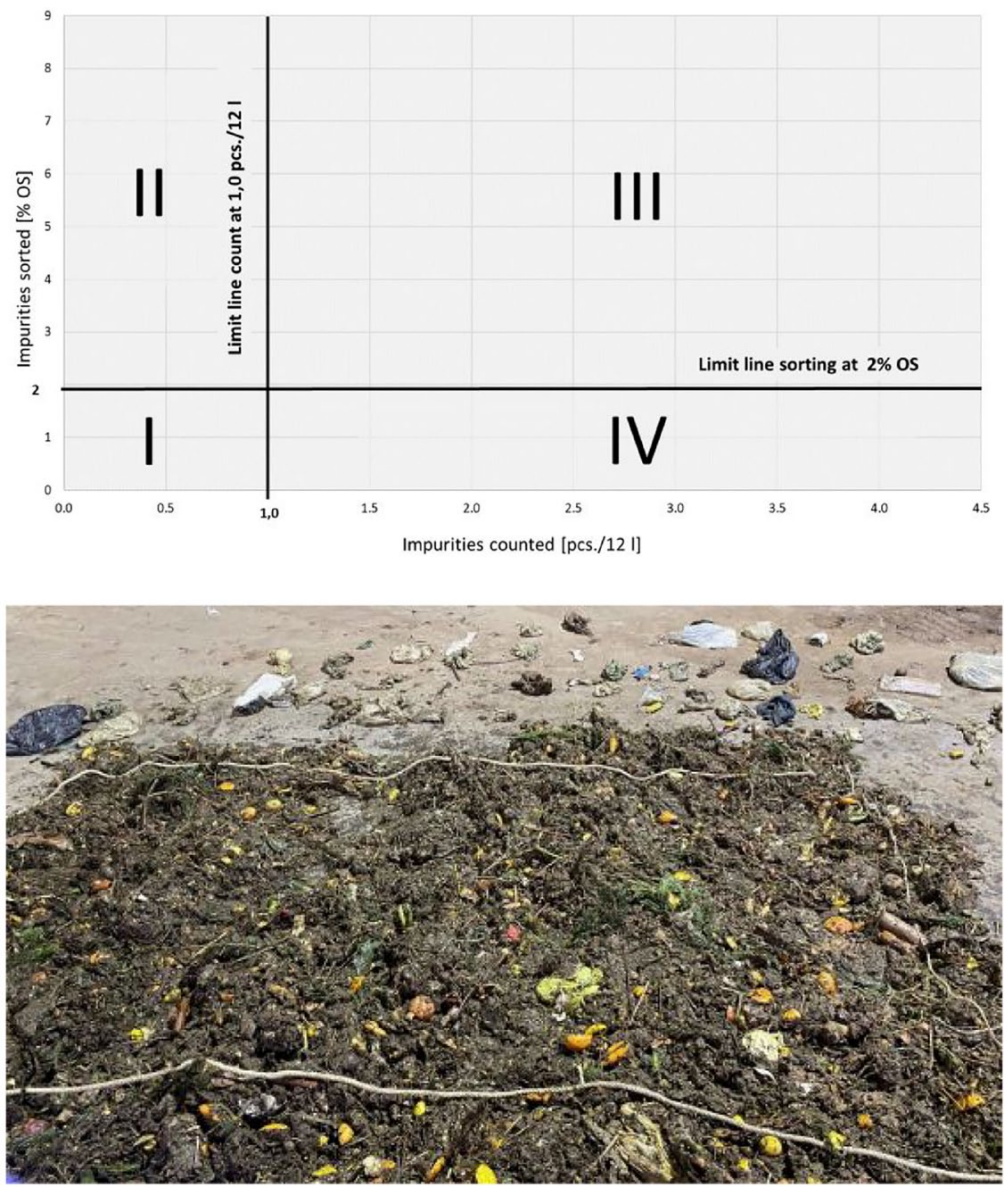
Above: presentation of the four possibilities for allocation to the dirty route at a limit value of 2 wt%_OS_ related to pcs./12 l, below: vehicle contents spread out for impurity counting.

The next step was to define four different possibilities in the following categories or quadrants to classify the respective bio-waste bins according to the degree of contamination (cf. Figure 1, top).

I. Not on the dirty route: below the limit value for actual impurities; below the limit value for counted impurities.
II. Actually on the dirty route: limit value of actual contaminants exceeded; limit value of counted contaminants not reached.
III. Rightly on the dirty route: limit value of actual impurities exceeded; limit value of the counted impurities exceeded.
IV. Wrongly on the dirty route: below the limit value for actual impurities; above the limit value for counted impurities.

The limit value for actual impurity content is 2 wt%_OS_, that is, the value corresponding to the limit value for the input material in the composting plant in accordance with the draft of the Compost Ordinance. Depending on the correlation, this results in a limit value for impurities counted. In Figure 1, top, this was set at 1 piece/12 l.

#### Rapid measurement method for routes (vehicle level)

In order to be able to evaluate entire vehicle contents in terms of contaminant content when they deliver to composting plants more easily and as time-efficiently as possible, work was also carried out at vehicle level to develop a practical rapid measurement method using contaminant counting.

In this process, the vehicle contents were dumped, mixed using the wheel loader, adapted to the desired sample quantity by quartering, and at least two samples of 500 kg each were taken. The samples were spread on the ground by a wheel loader to a height of approximately 20 cm. A rectangle with an area of 8 m^2^ was then framed by a rope on the spread-out waste, for example, 4 × 2 m (cf. Figure 1, below), or on an alternative area that is as large as possible. The interfering materials visible within the rectangle were counted. This process was repeated once or twice after the material was mixed by the wheel loader and then spread out again.

#### Areas analysed

A total of 10 areas in the province of Styria were investigated in the period from February 2019 to October 2022 (cf. Table 2). The areas were selected due to their geographical location and the variety of different demographics with a high degree of similarity in collection and separation behaviour. In addition, the great importance of the issue of impurities in bio-waste from household collection played a significant role in the selection of these areas. Individual municipalities were categorised based on the population density (PD) in inhabitants per km^2^ and divided into the three:

1. Rural area: 0−75 cap./km^2^ = 3 areas.
2. Small town area: 75−1000 cap./km^2^ = 5 areas.
3. Urban area: 1000−10,000 cap./km^2^ = 2 areas.

**Table 2.** Areas investigated.

Sort. No.	Time	Area	Category	Population density (cap./km^2^)	Bio-waste generation (kg/IN)	Number of bins	Quantity sorted (kg)	Focus investigation
1	February 2019	Area 1	Urban	3244	75	20	599	Determination of actual content of impurities with focus on multi-party buildings
2	February 2019	Area 2	Urban	8652	75	20	392	
3	February 2019	Area 3	Small town	185	66	20	598	
4	February 2019	Area 4	Small town	277	69	20	895	
5	February 2019	Area 5	Small town	166	43	20	1090	
6	December 2020	Area 1	Urban	3186	82	21	527	Assessment method at tonne level (pcs./12 l), impact measures
7	December 2020	Area 2	Urban	8532	82	20	297	
8	December 2020	Area 4	Small town	275	77	36	1787	
9	December 2020	Area 5	Small town	162	46	31	1718	
10	March 2021	Area 4	Small town	273	75	Pile	3860	Content of impurities
11	December 2021	Area 1	Urban	3022	77	13	161	Valuation method on tonne level (pcs./m^2^), Long-term effect measures
12	December 2021	Area 2	Urban	8342	77	19	401	
13	December 2021	Area 6	Rural	45	48	Tour	3180	Evaluation method at vehicle level (pcs./m^2^) Use of pre-gathering aids
14	December 2021	Area 7	Small town	90	100	Tour	2000	
15	December 2021	Area 8	Rural	58	93	Tour	1850	
16	March 2022	Area 4	Small town	270	74	Tour	1270	
17	March 2022	Area 5	Small town	159	43	Tour	1400	
18	May 2022	Area 9	Small town	913	32	59	1313	Use pre-gathering aids
19	May 2022	Area 10	Rural	18	29	40	363	
20	October 2022	Area 9	Small town	913	32	59	928	
21	October 2022	Area 10	Rural	18	29	40	585	

Population density is the number of inhabitants in relation to total area. No different criteria were established. Similar conditions can be assumed with regard to income, level of education and proportion of foreigners. The individual categories are characterised by different settlement structures. In the urban and small-town categories, the analyses were mainly carried out on blocks of flats. In the rural areas, mainly single and two-family houses were observed. The data were provided by the responsible municipal authorities or the respective waste management associations.

Manual sorting to determine impurities was used throughout the research. It served as a reference method for the development of the rapid measurement methods per tonne and per vehicle.

### Measures to influence the collection quality of separately collected bio-waste

The testing of the effect of individual measures for behavioural change or for the reduction of mis-sorting by citizens in bio-waste disposal was carried out in study areas 1, 2, 4 and 5. Problematic collection routes in terms of contaminant content, including blocks of flats, were selected. Each route chosen included 20 properties with varying numbers of 120 or 240 l bio-waste bins.

**Figure 2.**
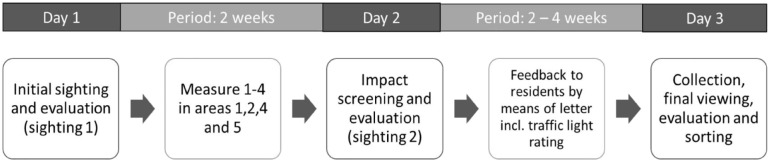
Sequence plan of measures and examinations to influence collection quality.

Figure 2 shows an overview of the research procedure to influence the collection quality of separately collected bio-waste. First of all, an initial triage (triage 1) was carried out to evaluate the initial situation.

Then, the following four measures were established for the properties:

- Measure 1 in area 1: Combination of a letter and the threat of a special emptying fee with the distribution of paper bags and waste separation sheets.
- Measure 2 in area 2: Letter with the threat of a special emptying fee or transfer to a dirt tour.
- Measure 3 in area 4: Distribution of paper bags with a cover letter and separation guide.
- Measure 4 in area 5: Motivational letter with waste separation sheets.

In this process, personally addressed letters with different content, with or without a series of paper bags, were distributed to each residential party, partly through the mailboxes, partly personally, partly by placing them at the front door. The cover letter indicated that the bio-waste bins would be inspected in the next few weeks. Following the inspection (examination 2) after 2 weeks, feedback using traffic light system was given to all of the inhabitants in a cover letter, once again with the respective evaluation. During the third triage another 2–4 weeks later, the bio-waste bins from each property were collected individually. Each of the bio-waste bins from each of the 20 properties was evaluated after being poured onto the sorting table using the visual counting method, and then each bin was sorted by hand. The sorting results were summarised to present the area values and output as a total value.

The studies were conducted over a total period of 4–6 weeks during the winter, that is, a short-term effect was measured.

Visual assessment with impurity counting was used as the method for evaluating the quality of the individual bio-waste bins for all siftings (initial, impact and final sifting). On the basis of an initial surface inspection, including determination of the degree to which the bio-waste bins were filled, the number of impurities (examination A, number A) was determined. After the material was poured into the collection vehicle hopper or onto the sorting table, the impurities were counted (sighting B, number B). The interfering materials were counted together, with parts found in both triage A and triage B counted as only one interfering material.

The evaluation was based on a traffic light system. The number of misses counted was compared with the fill level determined. The limit of an evaluation between orange and red was one piece of incorrect waste per 20% filling volume. If this was exceeded, the respective property received a red rating. In the feedback letters to the residents, the following meanings were assigned to the individual traffic light colours:

Red: There are too many contaminants in the bio-waste = special emptying/dirty route.
Orange: The number of interfering substances in the bio-waste is acceptable. However, there is potential for improvement and, if the situation worsens, special emptying may be required.
Green: There are no impurities in the bio-waste, the collection operates correctly.

## Results and discussion

### Content and type of impurities

The total impurity content in the areas studied in the period from 2019 to 2022 ranged from 0.1 to 8.4 wt%_OS_. A similar range can also be seen in section ‘Introduction’ for municipal deliveries in Germany, where the impurity content ranges from 1.4 to 9.1 wt%_OS_. While the rural communities showed low and unproblematic impurity content with a mean value of 0.4 wt%_OS_, the small town and urban regions showed high problematic values with a mean value of 2.4 and 2.5 wt%_OS_. These values, as well as the overall mean value of all analyses of 2.1 wt%_OS_, are in the range of the assumed mean of 2–3 wt%_OS_ by weight (Kehres, 2021).

The reasons for the higher impurity content in urban areas can be seen as greater anonymity combined with poorer separation behaviour, as well as the longer distance to the organic waste bin and the associated use of plastic bags as pre-collection bags. In rural areas with mainly single-family homes, pre-collection takes place more frequently in reusable containers, and there is a higher level of awareness when it comes to separation. It is generally known that there is a disparity in waste separation behaviour between rural and urban areas. The residual waste analysis in Styria showed that 38% of residual waste in the city of Graz was bio-waste, whereas the proportion in rural areas was only 26% (The Styrian Government, 2019). The main reason for this is the use of one bio-waste bin by several residents, especially in apartment blocks, combined with the greater anonymity. In rural areas, this factor is absent, as an bio-waste bin is usually clearly assigned to a household.

**Figure 3.**
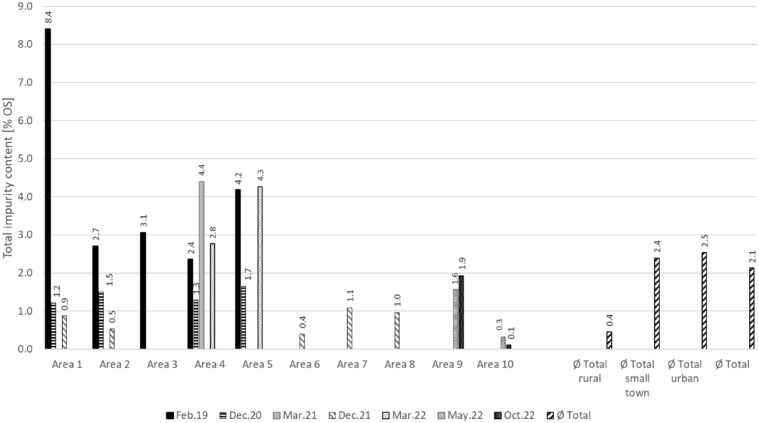
Impurity contents of sorting analyses 2019−2022 in 10 areas investigated, with mean values for rural, small town and urban areas, including totals.

In some areas with special measures, it was possible to reduce the impurity content, in others the measures had no effect (cf. Figure 3). The effect of the individual measures is described in detail in section ‘Influence of Different Measures on the Impurity Content in Bio-Waste’.

**Figure 4.**
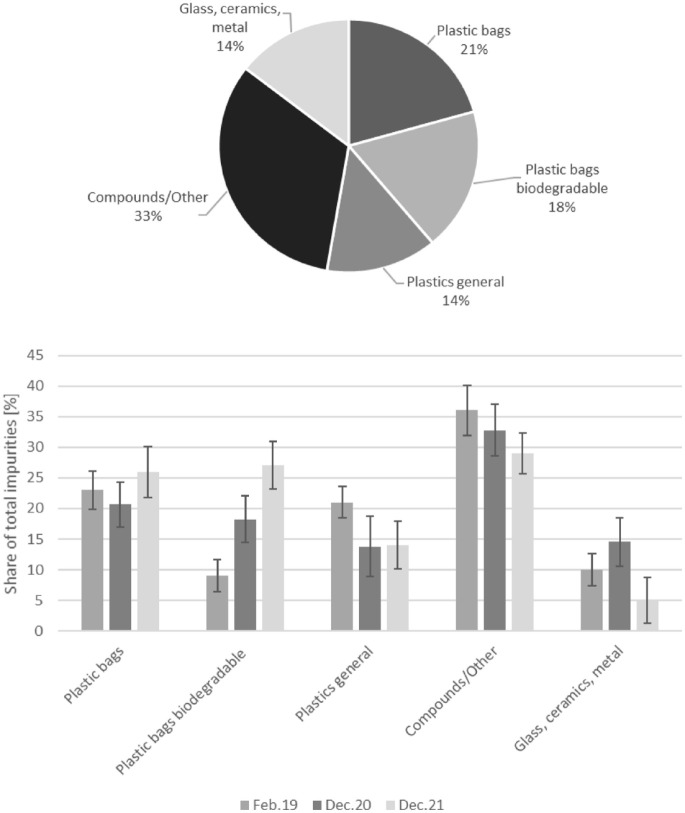
Above: breakdown of interfering substances in bio-waste in 120 bio-waste bins in December 2020 into 5 interfering substance groups as a percentage of the original substance, below: change in the proportions of 5 impurity groups in the total mass of impurities at 3 different points in time in 40−100 bio-waste bins each (measured as a percentage of original substance); February 2019 *n* = 100, December 2020 *n* = 80, December 2021 *n* = 4.

The impurities found were predominantly plastics. In a measurement in 120 bio-waste bins in December 2020, the share was 51%; when composites/others were included, the share rose to 86%. The sum of plastics could be further divided into bags and non-bags, the former into non-degradable (21%) and degradable (18%) and the latter into pure plastics (non-bags; 14%) and composites/others (33%). If plastic bags are combined, they account for the highest percentage among plastics. The remaining interfering materials (glass, ceramics, metals) played a subordinate role at 14% (Figure 4, top). An analysis of bio-waste in Germany also identified plastics as a clearly dominant factor with a share of 79% (Bundesgütegemeinschaft Kompost e.V., 2023). Sources in the literature often state the total impurity content in bio-waste. There is no separation into individual types of impurities.

Changes in the shares of the contaminant groups were observed throughout three measurement periods from 2019 to 2021. The share of biodegradable plastic bags increased, while the share of composites/other and glass/ceramics/metal decreased (Figure 4, below). The increase in bags with pre-collection bags made of plastic and biodegradable plastics is due to the growing supply combined with advertising in the retail sector. In addition, the use of pre-collection bags for bio-waste in private households makes disposal easier.

To estimate the confidence intervals, the relative standard deviation was calculated from the mean values of the individual analyses and the standard deviation. As the material is very inhomogeneous, it is not possible to determine the exact confidence intervals.

### Development of the rapid measurement method

#### Rapid measurement method at bio bin level

##### Correlation of volume-based count values with sorting results

**Figure 5.**
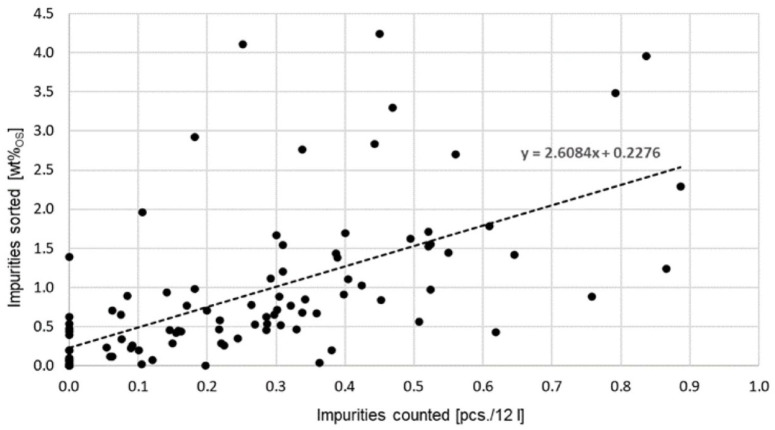
Correlation of the quick measurement method for organic bin contents with sorting results in 2020 using volume-based count values (*r* = 0.61, *n* = 101).

For 101 bio-waste bins examined in 4 selected areas (areas 1,2, 4 and 5) in 2020, the correlation coefficient between the impurities counted in pieces per 12 l in the bulk and the sorting results in wt%_OS_ is *r* = 0.61. This results in a satisfactory correlation of the new method with the sorting results (Figure 5).

When looking specifically at the correlation of the individual areas, the highest correlation coefficient of *r* = 0.75 was seen in areas 1 and 2 (*n* = 41 bio-waste bins). The correlation in area 5 was *r* = 0.52 (*n* = 31 bio-waste bins) and in area 4 was *r* = 0.38 (*n* = 36 bio-waste bins). The reason for these low correlations is the reference to volume. Higher correlations were found in the measurements based on surface areas. The effort for the quick measurement method presented was around 4 minutes for the contents of one bio-waste bin, which is 16% of the time needed for sorting.

##### Correlation of area-based count values with sorting results

**Figure 6.**
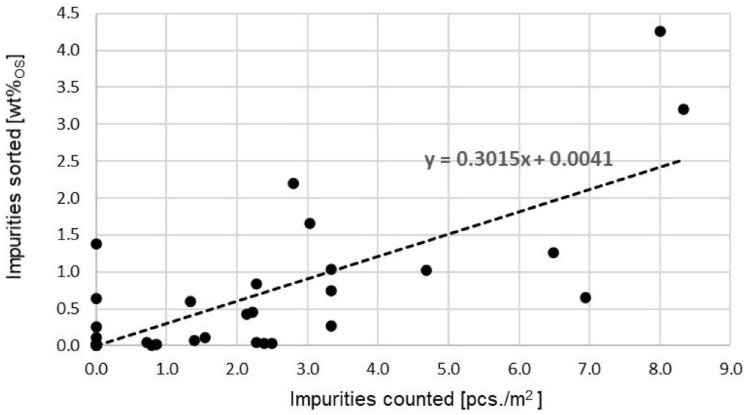
Correlation of the quick measurement method for organic bin contents with sorting results in 2021 using area-based count values (*r* = 0.74, *n* = 32).

The fact that 32 tonnes was examined in areas 1 and 2 results in a better correlation coefficient of *r* = 0.74 (Figure 6) for the area relationship of impurities counted, in pieces per square metre, to impurity content sorted.

#### Rapid measurement method at collection vehicle level

**Figure 7.**
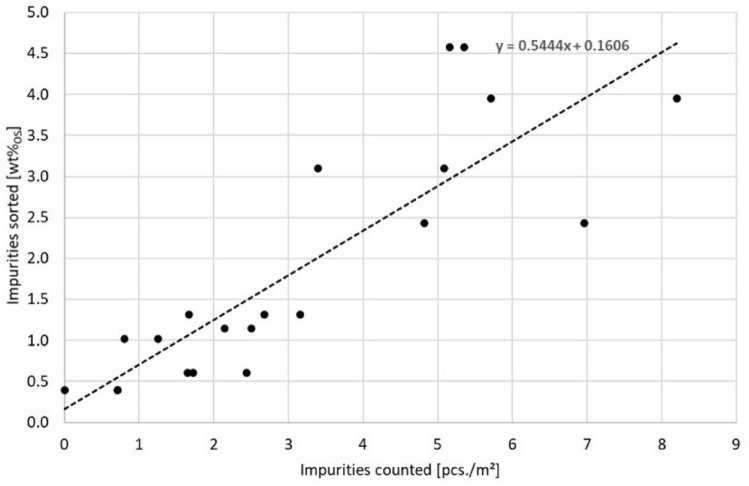
Correlation between the rapid measurement method for vehicle contents and the sorting results (*r* = 0.85, *N* = 20).

The rapid measurement method using impurity counting of vehicle contents was undertaken only with impurities counted per area in m^2^. Two to three samples were counted per area, each covering 8 m^2^. The number of pieces of interfering material taken was calculated on the area of 1 m^2^ for the determination of the correlation coefficient. The correlation coefficient is *r* = 0.85 (Figure 7) for areas 4 to 8 from the sorting analyses 13−17. The vehicles analysed were mainly press vehicles.

Each of the points in the diagram comprises the quick measurement of a vehicle’s contents and the subsequent sorting to determine the actual impurity content in the respective vehicle.

The better correlation at vehicle level compared to bio-waste bin level can be explained by the larger area considered for the vehicle contents. This results in higher counting results with the same contaminant load. The effort for the rapid measurement method presents was 20 minutes for the total vehicle content, which is around 8% of the time required for sorting.

### Influence of different measures on the impurity content in bio-waste

**Figure 8.**
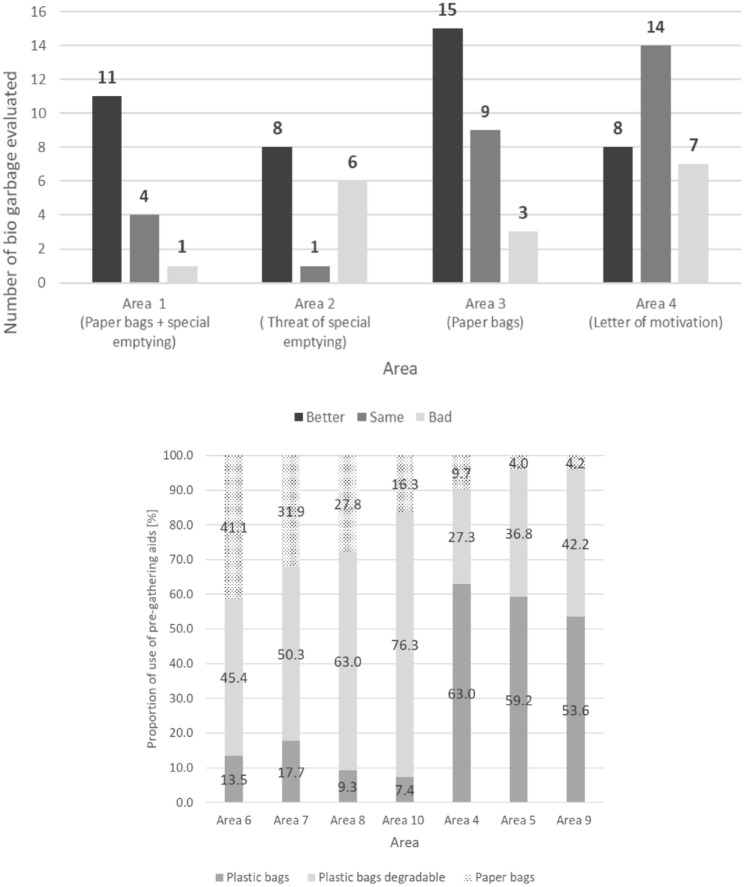
Above: effect of measures to improve the impurity content verified by the rapid measurement method at the level of bio-waste bins, below: proportions of pre-collection bags found by three groups studied.

The testing using the rapid measurement method of the effect of measures to reduce the impurity content on the bio-waste bins level was successful. Measures with an effect on the impurity content and ineffective measures were found. Significance was found for the former, as the vast majority of the bio-waste bins tested and evaluable resulted in improvement; the next lowest proportion resulted in neither improvement nor deterioration; and the lowest proportion resulted in deterioration. This effect occurred in two out of four areas where the same measure, distribution of paper bags, was applied. Accordingly, in area 1, where an additional cost increase was threatened for special emptying, an improvement in collection quality was achieved in 11 out of 16 bio-waste bins, and in area 3 in 15 out of 27 bio-waste bins examined. In the other two other areas, 2 and 4, where only a cost increase or only a motivational letter were used as measures, there were no significant effects (Figure 8, above).

In an investigation of the contaminant levels in two areas with the measures paper bag distribution and threat of a cost increase, the long-term effect could be proven 1 year after the measure. The impurity contents were then still only 0.5 and 0.9 wt%_OS_. On one hand, this may be due to the lasting effect of the measures taken combined with increased awareness. On the other hand, contaminated bio-waste may have been disposed of as residual waste.

That measures to improve the contaminant situation are not necessarily successful is shown by the result with the motivational letter alone, which did not result in an improvement of the situation. The distribution and promotion of biodegradable plastic bags also does not lead to an improvement in the contaminant situation (Figure 3, areas 9). The latter situation was also described by Arbeck et al. (2022). The reason for this could be the broken window effect. Citizens who are difficult to persuade to separate waste well tend to use non-degradable plastic bags as a pre-collection bag if their neighbours have their waste in plastic bags, even if these are degradable.

The rapid measurement method at vehicle level presented allows rapid determination of the effect of measures with little effort. This approach has been recommended for many years (Bundesgütegemeinschaft Kompost e.V., 2016; Flemke, 1997) but has rarely been implemented so far due to the great effort involved.

### Pre-collection bags

In rural areas, biodegradable plastic bags were predominant among the three pre-collection bags evaluated, accounting for 45−76% of the bags found. In urban areas, conventional plastic bags are predominant, with 54−63%. The reason is the awareness and acceptance by the rural population of biodegradable products in general. Costs can also be a factor. The unit costs for pre-collection bags made of biodegradable plastics are around 10 times higher than conventional plastic bags. Paper bags are used more in rural areas, at 16−41%, although only areas where paper bags are advertised and made available for purchase or free drop-off were recorded here (Figure 8, below).

### Positive improvements

It is possible to improve the quality of organic waste through measures such as the personal distribution of paper bags and penalties. The continuity of the measures, regular monitoring and communication are important here. Inspections of the organic waste bins have to be explained to citizens and penalty measures have to be accompanied by targeted public relations work.

### Instructions for using the quick measurement method in practice

Vehicle contents with collected bio-waste are unloaded and pulled apart using a wheel loader and spread out evenly flat at a height of at least 300 mm. The ground near the area to be assessed should not be visible. Then a specific assessment area of, for example, 8 m^2^ is staked off with a rope and the number of recognisable impurities on the surface is determined. This produces a surface load in pieces per square metre. The area counted varies depending on the load. The correlation graph can then be used to determine the impurity content.

## Conclusion

It was possible to obtain sufficiently reliable data on the type and quantity of contaminants in the areas investigated. The total impurity content ranged between 0.1 and 8.4 wt%_OS_. Plastics predominate among the interfering substances at 53%. Among these, pre-collection bags made of plastic accounted for the highest share. The studies confirmed an increase in the contaminant content with the population density. In recent years, there has been an increase in the proportion of biodegradable plastic bags.

It has been possible to prove the functionality of the two rapid measurement methods. Depending on the application, the methods developed reduce the staff and time required by around 80–90% compared to classic manual sorting. The correlation of the two methods is good, with a coefficient of 0.74, at bio-waste bin level and very good (0.85) at vehicle level. For this reason, the new method can be recommended for practical contaminant measurements in bio-waste for individual bins and for vehicle contents on the tour level. For the individual municipalities, rapid visual determination of the impurity content by an employee at bio-waste bin level leads to the possibility of clear identification of problematic properties and, if necessary, provides evidence for classification into a more costly “dirty collection”. If the simple opening of the lid of the bio-waste bin is sufficient for a decision on the resulting measure (e.g. a complaint), this should be preferred, as the effort is less, and satisfactory correlation with the actual impurity content has also been found here (Giani and Pretz, 2015).

Rapid determination at entire vehicle contents level creates a practicable and resource-efficient method that can be relied on by collectors and compost plant operators, on one hand, with regard to compliance with legal limit values and, on the other, as a basis for contractual regulations.

By identifying heavily contaminated routes and properties with poor motivation for separation, large-scale targeted measures can be taken that sustainably reduce the level of contaminants for the compost plant operator.

It was possible to test individual measures to change behaviour conclusively with the method used. The distribution of paper bags and the threat of a cost increase were found to be the measures most effective in reducing the level of impurities. In this case, it was possible to reduce the contaminant content in the short term, within 6 weeks. In one area, a long-term effect could also be detected after 1 year. A purely motivational approach was not successful.

With regard to the use of pre-collection bags, a higher proportion of biodegradable plastic bags is found in rural municipalities, whereas normal plastic bags predominate in more densely populated areas. Paper bags are only detectable in relevant quantities when actively promoted by the municipality. The background for this may also be the low supply of paper bags in local shops compared to degradable and non-degradable plastic bags.

The results demonstrate the advisability of rapid measurement methods to quickly determine the impurity content. Targeted measures over a longer period of time for properties and routes identified as problematic reduce the impurity content and are seen as target-oriented. There is a need for further research and the content of the measures reviewed was not studied.

For decision-makers in municipalities and associations, the results obtained and the individual methods are important tools with regard to future strategy and prioritisation in the area of biogenic waste collection from households.

## References

[bibr1-0734242X241259895] Arbeck N Kern M Siepenkothen J , et al. (2022) Praxistest Bio-Beutel – Kreislaufwirtschaft mit kompostierbaren Obst- und Gemüsebeuteln [Practical Testing of Bio-Waste Bags – Circular Economy with Compostable Fruit and Vegetable Bags]. C.A.R.M.E.N. e.V., Witzenhausen, Germany.

[bibr2-0734242X241259895] Bauer E (2017) Die Qualität der Bioabfallsammlung in Abhängigkeit von der Siedlungsstruktur und dem Sammelsystem im Bezirk Graz-Umgebung [The quality of biowaste collection depending on the settlement structure and the collection system in the district of Graz-Umgebung]. Masterarbeit, University of Graz, Graz, Austria.

[bibr3-0734242X241259895] Bund/Länder-Arbeitsgemeinschaft Abfall (LAGA) (2022) Getrenntsammlung von Bioabfällen [Separate Collection of Biowaste]. Bremen, Germany. https://www.laga-online.de/documents/anlage-1-aktual2023-bericht-laga-ag-getrenntsammlung-bioabfaelle-2022-stand-juni-2024_1723110027.pdf (accessed 13 August 2024).

[bibr4-0734242X241259895] Bundesgesetz (1992) Verordnung des Bundesministers für Umwelt, Jugend und Familie über die getrennte Sammlung biogener Abfälle [Ordinance of the Federal Minister for the Environment, Youth and Family Affairs on the Separate Collection of Bio-Waste]. RIS, Federal Law Gazette No. 68/1992. Vienna, Austria.

[bibr5-0734242X241259895] Bundesgütegemeinschaft Kompost e.V. (2016) Sortenreinheit von Bioabfällen gewährleisten [Ensuring the Purity of Biowaste]. Bundesgütegemeinschaft Kompost e.V., Cologne-Gremberghoven, Germany.

[bibr6-0734242X241259895] Bundesgütegemeinschaft Kompost e.V. (2018/2019) Untersuchung des Gehaltes an Fremdstoffen in angelieferten Bioabfällen mittels Chargenanalyse [Analysis of the Content of Foreign Substances in Delivered Biowaste by Means of Batch Analysis]. Bundesgütegemeinschaft Kompost e.V., Germany.

[bibr7-0734242X241259895] Bundesgütegemeinschaft Kompost e.V. (2021) Methodenbuch zur Analyse organischer Düngemittel, Bodenverbesserungsmittel und Substrate der BGK [Method for the Analysis of Organic Fertilisers, Soil Improvers and Substrates of the BGK]. Bundesgütegemeinschaft Kompost e.V., Köln-Gremberghoven, Germany.

[bibr8-0734242X241259895] Bundesgütegemeinschaft Kompost e.V. (2023) Fremdstoffanalyse von Biogut – Bericht zum BKG Workshop Sortenreinheit von Bioabfällen [Analysis of impurities in bio-waste – Report on the BKG workshop on the purity of bio-waste]. Bundesgütegemeinschaft Kompost e.V., Köln-Gremberghoven, Germany.

[bibr9-0734242X241259895] European Compost Network ECN e.V. (2022) ECN-rapport-2022.– COMPOST AND DIGESTATE FOR A CIRCULAR BIOECONOMY Overview of Bio-Waste Collection, Treatment & Markets Across Europe. Bochum, Germany. https://www.compostnetwork.info/wordpress/wp-content/uploads/ECN-rapport-2022.pdf (accessed 13 August 2024).

[bibr10-0734242X241259895] European Parlament (2018) Richtlinie (EU) 2018/ des Europäischen Parlaments und des Rates vom 30. Mai 2018 zur Änderung der Richtlinie 2008/98/EG [Directive (EU) 2018/ of the European Parliament and of the Council of 30 May 2018 amending Directive 2008/98/EC on waste]. über Abfälle, Brussels.

[bibr11-0734242X241259895] Federal Ministry for Climate Action, Environment, Energy, Mobility, Innovation and Technology (2021) Entwurf Kompostverordnung [Draft compost ordinance] (unpublished version). Vienna, Austria.

[bibr12-0734242X241259895] Federal Ministry for Climate Action, Environment, Energy, Mobility, Innovation and Technology (2023) Bundes-Abfallwirtschaftsplan 2023 [Federal waste management plan 2023]. Vienna, Austria. https://www.bmk.gv.at/themen/klima_umwelt/abfall/aws/bundes_awp/bawp2023.html (accessed 13 August 2024).

[bibr13-0734242X241259895] Flemke H (1997) Vermeidung von Störstoffeinträgen in die Biotonne [Avoiding contaminants in the organic waste bin]. In: Abfallwirtschafsjournal 9, EF-Verl. für Energie u. Umwelttechnik, Berlin, pp.20–27.

[bibr14-0734242X241259895] Giani H Pretz T (2015) Qualitätsoffensive 20 Jahre Biotonne in der Stadt Würselen [Quality campaign 20 years of organic waste bins in the town of Würselen], I.A.R. Institut für Aufbereitung und Recycling, Aachen, Germany.

[bibr15-0734242X241259895] Habermann P-M (2022) Innovative Öffentlichkeitsarbeit und Bioabfallaufbereitung zur Sicherstellung einer hochwertigen Kompostqualität [Innovative public relations work and biowaste processing to ensure high-quality compost]. Bundesgütegemeinschaft Kompost e. V., Witzenhausen, Germany.

[bibr16-0734242X241259895] Idelmann M Werk S Abbing M. (2022) Störstoffreie Biotonne durch Verbraucherkommunikation und Tonnenkontrollen mit der geodatenbasierten Handy-App [Contaminant-free bio-waste bins through consumer communication and bin checks with the geodata-based mobile phone app]. Bundesgütegemeinschaft Kompost e. V., Witzenhausen, Germany.

[bibr17-0734242X241259895] Jansen C Brune M Pretz T , et al. (2020) Energieeffiziente Bioabfallbehandlung durch Konditionierung – EnKoBio [Energy-efficient biowaste treatment through conditioning – EnKoBio]. RWTH Aachen University, Schlussbericht (Final report), Germany.

[bibr18-0734242X241259895] Jörg B (2019) Qualitätssicherung bei der Bioabfallbehandlung [Quality assurance in biowaste treatment]. Bioabfallforum Baden-Württemberg 2019, Vortrag (Lecture). Germany.

[bibr19-0734242X241259895] Kehres B (2017) Problem Fremdstoffe/Kunststoff in Bioabfall und Kompost [Problem of foreign matter/plastic in biowaste and compost]. Bundesgütegemeinschaft Kompost e. V., Germany.

[bibr20-0734242X241259895] Kehres B (2018) Gebietsanalyse Bestimmung der Sortenreinheit von Biogut eines Entsorgungsgebietes [Area analysis determination of the varietal purity of biowaste in a disposal area]. Bundesgütegemeinschaft Kompost e. V., Germany.

[bibr21-0734242X241259895] Kehres B (2021) Leitfaden zu einem Qualitätsmanagement der sortenreinen Bioguterfassung [Guidelines for quality management of single-variety bio-waste collection]. Bundesgütegemeinschaft Kompost e. V., Witzenhausen, Germany.

[bibr22-0734242X241259895] Kehres B Thelen-Jüngling M (2021) Chargenanalyse – Methode zur Bestimmung des Fremdstoffgehaltes fester Bioabfälle [Batch analysis – Method for determining the foreign matter content of solid biowaste]. Bundesgütegemeinschaft Kompost e.V., Germany.

[bibr23-0734242X241259895] Kern M Siepenkothen J Neumann F (2017) BiogutRADAR – Bonitierung von Biotonnen zur Prognose von Fremdstoffgehalten im Biogut [BiogutRADAR – Scoring of organic waste bins to predict the content of foreign matter in organic waste]. In: Müll & Abfall 6/17. Erich Schmidt Verlag GmbH & Co. KG, Berlin, pp.287–291.

[bibr24-0734242X241259895] Kompostproduzent Steiermark (2020) Daten Anlieferung und Entsorgung Bioabfall 2020. Persönliche Nachricht, Styria, Austria.

[bibr25-0734242X241259895] ÖWAV (2021) Österreichischer Wasser- und Abfallwirtschaftsverband. Expertenpapier – Bio-Kunststoffe und die biologische Abfallverwertung (Expert paper – Bioplastics and biological waste utilisation). Vienna, Austria. https://www.oewav.at/Kontext/WebService/SecureFileAccess.aspx?fileguid={6cb724ce-71df-4216-95f2-e912b6d444cb} (accessed 13 August 2024).

[bibr26-0734242X241259895] Reichel E (2020) Bio-waste in Europe. Turning challenges into opportunities, No. 2020, 04: Publications Office of the European Union, Luxembourg.

[bibr27-0734242X241259895] Ricci MJ Centemero M (2018) Separate collection of food waste and rejects. In: Müll und Abfall. Erich Schmidt Verlag GmbH & Co. KG, Berlin, pp.340–343.

[bibr28-0734242X241259895] The Styrian Government (2019) Restmüllanalyse im Land Steiermark 2018/19 [Analyses of residual waste in the province of styria 2018/19]. Graz, Austria. https://www.abfallwirtschaft.steiermark.at/cms/beitrag/12737874/134974365/ (accessed 13 August 2024).

[bibr29-0734242X241259895] Wellacher M Krenn A (2017) Störstoffanalyse in der Biotonne im Stadtgebiet von Leoben (Analysis of impurities in the bio-waste bin in the city of Leoben). Leoben, Austria. https://www.ibwellacher.at/wp-content/uploads/2021/01/Vortrag-Hannover-2017.pdf (accessed 13 August 2024).

